# Electrical output of bryophyte microbial fuel cell systems is sufficient to power a radio or an environmental sensor

**DOI:** 10.1098/rsos.160249

**Published:** 2016-10-26

**Authors:** Paolo Bombelli, Ross J. Dennis, Fabienne Felder, Matt B. Cooper, Durgaprasad Madras Rajaraman Iyer, Jessica Royles, Susan T. L. Harrison, Alison G. Smith, C. Jill Harrison, Christopher J. Howe

**Affiliations:** 1 Department of Biochemistry, University of Cambridge, Hopkins Building, Downing Site, Tennis Court Road, Cambridge CB2 1QW, UK; 2 Department of Plant Sciences, University of Cambridge, Downing Site, Downing Street, Cambridge CB2 3EA, UK; 3 Department of Chemical Engineering, Centre for Bioprocess Engineering Research, University of Cape Town, Rondebosch 7701, Cape Town, South Africa; 4 School of Biological Sciences, University of Bristol, Life Sciences Building, Downing, 24 Tyndall Avenue, Bristol BS8 1TQ, UK; 5 The Commonwealth Scientific and Industrial Research Organisation (CSIRO), Division of Plant Industry, Canberra, Queensland, Australia

**Keywords:** microbial fuel cell, plant microbial fuel cell, bioelectricity, electrochemistry, bryophyte, bryoMFC

## Abstract

Plant microbial fuel cells are a recently developed technology that exploits photosynthesis in vascular plants by harnessing solar energy and generating electrical power. In this study, the model moss species *Physcomitrella patens*, and other environmental samples of mosses, have been used to develop a non-vascular bryophyte microbial fuel cell (bryoMFC). A novel three-dimensional anodic matrix was successfully created and characterized and was further tested in a bryoMFC to determine the capacity of mosses to generate electrical power. The importance of anodophilic microorganisms in the bryoMFC was also determined. It was found that the non-sterile bryoMFCs operated with *P. patens* delivered over an order of magnitude higher peak power output (2.6 ± 0.6 µW m^−2^) than bryoMFCs kept in near-sterile conditions (0.2 ± 0.1 µW m^−2^). These results confirm the importance of the microbial populations for delivering electrons to the anode in a bryoMFC. When the bryoMFCs were operated with environmental samples of moss (non-sterile) the peak power output reached 6.7 ± 0.6 mW m^−2^. The bryoMFCs operated with environmental samples of moss were able to power a commercial radio receiver or an environmental sensor (LCD desktop weather station).

## 1. Introduction

Population and economic growth drive global energy demand. World annual energy consumption is predicted to be approximately 8.59 × 10^20^ J in 2040—about 56% growth over 30 years [1]. Increasing demand has led to the development of alternative energy production technologies to alleviate concerns about the limited availability of fossil fuels [2].

Some of the existing alternatives to fossil fuels include nuclear [3,4], hydro-electric, geothermal and wind power, biomass generation [5] and solar power (e.g. photovoltaic panels), which are all predicted to play important roles in the years to come. While the use of these alternatives offers undoubted advantages such as reduced CO_2_ emissions and a renewable energy source, a number of disadvantages counterbalance those benefits, such as geographical limitations for hydro-electric and geothermal power, landscape transformation for wind power, use of arable land for the production of biomass and energy-intensive processes for the production of photovoltaic panels. It has therefore been estimated that fossil fuels will still provide almost 78% of world energy demand in 2040 [1]. Novel energy generation systems are therefore likely to be an important supplement to existing systems.

Some microorganisms, termed exoelectrogens, are known to oxidize organic substrates and donate electrons to conductive materials (e.g. electrodes) [6] and this phenomenon has previously been exploited in the development of microbial fuel cells (MFCs) [7]. Electron transfer to electrodes has been suggested to occur through direct mechanisms [8–10], such as membrane-bound electron shuttles [11–14], conductive nanowires [15–17] and/or mechanisms mediated through extracellular redox shuttles [18]. The electrons are directed through an electrical circuit to reduce an electron acceptor (e.g. O_2_) in the cathodic region. A similar principle is employed in vascular plant microbial fuel cells (PMFCs) and biophotovoltaics (BPVs) that use the light-harnessing ability of vascular plants and unicellular photosynthetic organisms, respectively, to generate electrons [10,19–23].

PMFCs use organic rhizodeposits, comprising root exudates and dead root cells, as the electron donor for heterotrophic microorganisms in the plant rhizosphere [23]. The phloem in vascular plants aids in the transport of photosynthates and secondary metabolites to plant roots [24]. It is therefore unclear how much power generation, if any, would be possible with photosynthetic organisms that lack vascular tissue.

The present investigation therefore serves to study the electrical output of bio-electrochemical systems operating with bryophytes (the laboratory strain *Physcomitrella patens* as well as environmental moss samples) as examples of photosynthetic organisms that lack vascular tissue. Bryophytes are poikilohydric plants with a greater tolerance to dehydration than vascular plants [25,26]. Bryophytes have a unique physiology enabling them to accumulate water and nutrients, and survive in a wide range of temperatures and habitats. They have root hair-like rhizoids that bind the surface on which they grow, stabilizing the soil and preventing loss of nutrients by erosion. Bryophytes provide a source of food for microbial consortia, and influence carbon and nitrogen cycling in the atmosphere [26]. *Physcomitrella patens* is widely used as model bryophyte; it has an erect growth habit forming small tufts. These tufts are 10–20 mm tall (electronic supplementary material, figure S1) and are formed by leafy shoots developing from a filamentous base. It has short, oblong to lanceolate leaves, to approximately 5 mm, with finely serrate margins, and the leaves are arranged in a spiral surrounding the apex [27].

**Figure 1.**
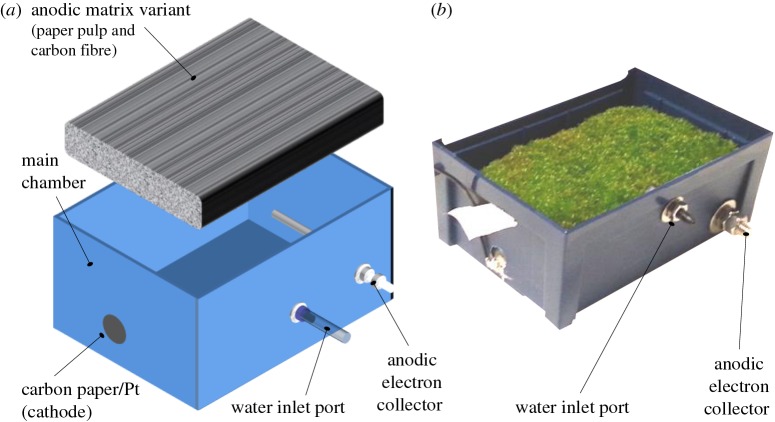
Construction of the bryoMFCs. (*a*) Semi-exploded three-dimensional cartoon of the bryoMFC system. (*b*) BryoMFC device with an established culture of *P. patens*.

The first bryophyte microbial fuel cell (bryoMFC), based on the forest moss *Dicranum montanum*, was reported at the First International PlantPower Symposium in Ghent, Belgium in 2011 [28]. In this study, bryoMFCs (figure 1) have been further developed and investigated in more detail. To the best of our knowledge, this is the first published study of an MFC operated with the bryophyte *P. patens*. The bryoMFCs were constructed with a novel three-dimensional anodic matrix (electronic supplementary material, figure S2) that has electrically conductive carbon fibres throughout the entire volume of the matrix. This was used to enhance physical contact between moss tissues and the anodic surface. This study also compared the electrical output of bryoMFCs operated in non-sterile conditions with systems operated in near-sterile conditions to clarify the role of the anodophilic microbes for energy transduction in bryoMFCs. Finally, the electrochemical device developed for testing *P. patens* was operated with environmental samples of moss to power a commercial radio or a LCD desktop weather station.

## 2. Results

### 2.1. Anodic matrix

**Figure 2.**
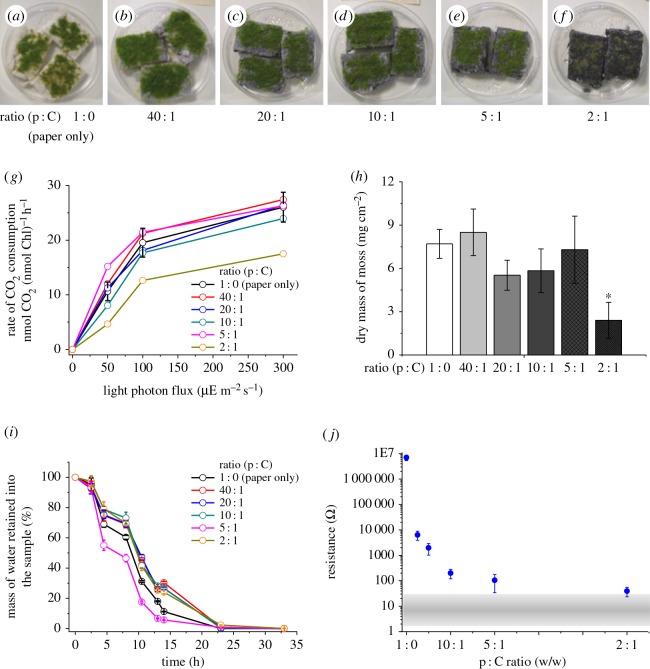
Characteristics of matrix variants. The figure shows the characteristics of the anodic matrix variants and the control with established cultures of *P. patens* as follows: (*a*–*f*) samples of anodic matrix variant. Paper to carbon fibre (p : C) as weight-to-weight varied from 1 : 0 (control) (*a*), 40 : 1 (*b*), 20 : 1 (*c*), 10 : 1 (*d*), 5 : 1 (*e*) to 2 : 1 (*f*). (*g*) Rate of CO_2_ consumption measured for *P. patens* (*n* = 3 for the control sample and *n* = 1 for samples of anodic matrix variant). (*h*) Biomass accumulation over 20 days for *P. patens* grown on the control sample and samples of anodic matrix variant (*n* = 3 for the first four samples and *n* = 2 for the last two samples). Asterisk, ANOVA test found the value differed significantly from the group of samples containing the control (p : C ratio 1 : 0) with a *p*-value of less than or equal to 5%. (*i*) Rate of mass lost owing to water evaporation for the control and samples of anodic matrix variant (*n* = 3 for all the samples). (*j*) Electric resistance for the control sample and samples of anodic matrix variant (of the same size) and a planar sheet of carbon fibre (grey bar; *n* = 3 for all the samples).

A novel matrix was created and used in the bryoMFCs as an anodic electron conductive substrate. This matrix was made by integrating paper and carbon fibre as described in the Material and methods section and shown in the electronic supplementary material, figure S2. A range of matrices was constructed with different ratios of paper to carbon fibre as well as a sample made with paper only. To serve as an anodic substrate for a bryoMFC, the matrix has to have several characteristics such as: (i) biocompatibility, (ii) water retention, and (iii) low electric resistance. The compatibility of *P. patens* with this anodic matrix was tested. The results are illustrated in figure 2*a*–*j*. *Physcomitrella patens* grew on each of the six samples tested. Nevertheless, *P. patens* grown on the matrix variant with the highest load of carbon fibre (paper to carbon fibre (p : C) ratio 2 : 1) appeared to be smaller. This qualitative observation was verified by measuring the rate of carbon consumption and biomass accumulation for *P. patens* grown on the matrix variants. The CO_2_ consumption rate of *P. patens* grown on the matrix variants indicated that, with the exception of the sample with the highest loading of carbon (p : C ratio 2 : 1), rates were not dissimilar to the CO_2_ consumption rate observed for plants grown on the paper-only reference control (figure 2*g*). In addition, other than the sample with the highest loading of carbon (p : C ratio 2 : 1), biomass accumulation values on the other four samples were not dissimilar to the reference control (figure 2*h*).

A suitable anodic matrix should be able to retain water over time, for plant growth and for conductivity in the electrochemical systems—a dry anodic matrix would be a severe impediment for charge movement within the anodic chamber. We therefore measured the rate of mass loss due to water evaporation over a period of 30 h after the matrix variants had been hydrated with 50 ml of deionized water for 10 g of dry mass (figure 2*i*). Based on water loss from the matrix over the first 10 h, we found that the five specimens of matrix variants and the control had similar water retention characteristics.

The final parameter tested was electrical resistance. A planar sheet of carbon fibre was also tested (p : C ratio 0 : 1) along with the control sample and the five matrix variants. The paper control sample (p : C ratio 1 : 0) showed maximum electrical resistance of 6.97 ± 1.72 MΩ. The five samples of matrix variant with increased loading of carbon fibre showed lower resistance (figure 2*j*). For the planar sheet of carbon fibre (p : C ratio 0 : 1), the resistance measured ranged between 5 and 20 Ω as shown in figure 2*j* (grey horizontal strip). Taking these observations together, a ratio p : C of 10 : 1 was chosen to conduct the experimental work presented below.

### 2.2. Trends in electric output of bryophyte microbial fuel cells

**Figure 3.**
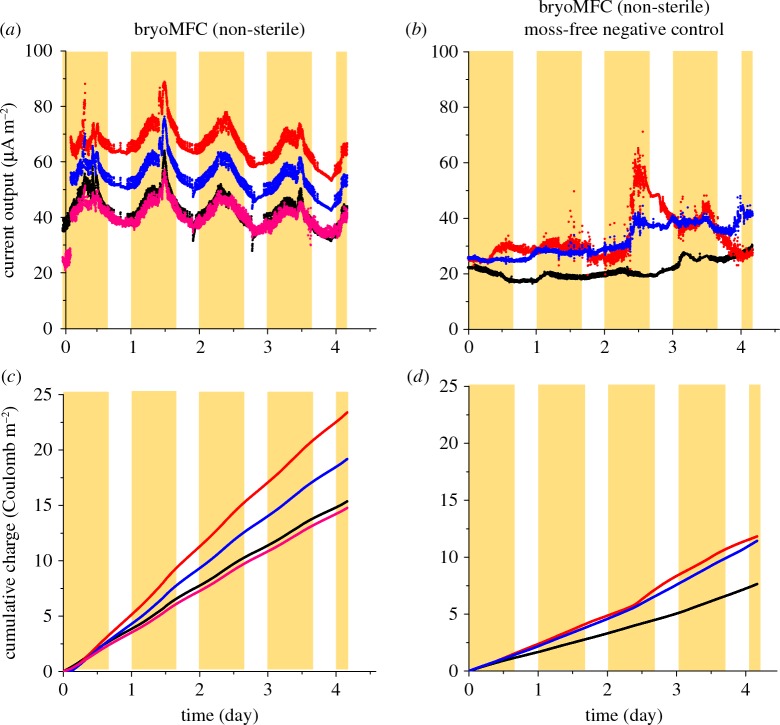
Current output and charge accumulation in non-sterile bryoMFCs. The figure shows continuous records of current output (*a,b*) and total charge accumulation (*c,d*) with a fixed external load of 4.6 kΩ for *P. patens* and negative control, respectively, in bryoMFCs (non-sterile). The yellow background shows the phases with light incident on the bryoMFCs (*n* = 4 for the non-sterile bryoMFC and *n* = 3 for the negative controls).

**Figure 4.**
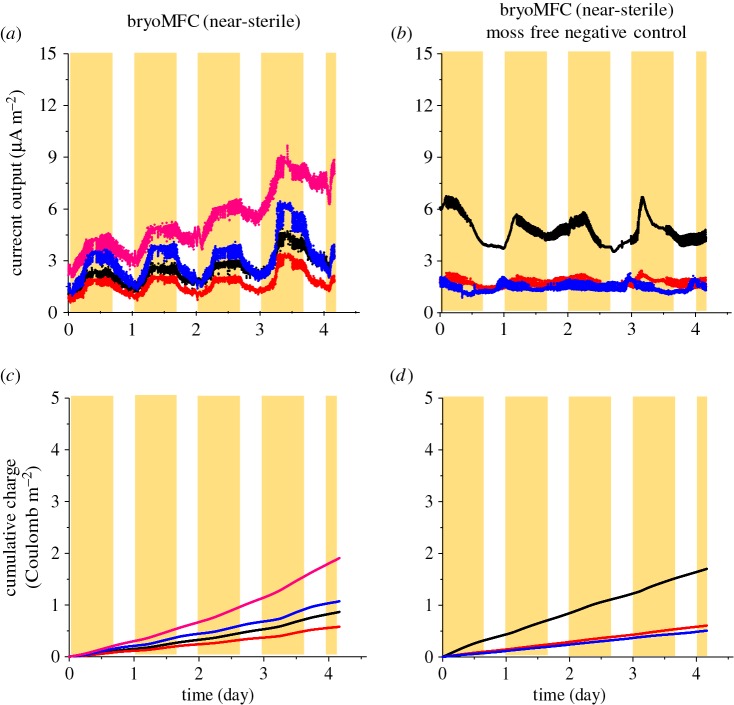
Current output and charge accumulation in near-sterile bryoMFCs. The figure shows continuous records of current output (*a,b*) and total charge accumulation (*c,d*) with a fixed external load of 4.6 kΩ for *P. patens* and negative control, respectively, in bryoMFCs (near-sterile). The yellow background shows the phases with light (*n* = 4 for the near-sterile bryoMFC and *n* = 3 for the negative controls).

The bryoMFCs using the novel anodic matrix described above were sterilized by autoclaving and then inoculated with a protonemal *P. patens* homogenate. One set was kept in non-sterile conditions and compared with bryoMFCs inoculated and maintained in near-sterile conditions. The near-sterile systems were kept closed throughout the experimental run to minimize microbial contamination, and water was provided via a sterile filter. The features of the devices are described in the Material and methods section and shown in figure 1. Current density was calculated from the voltage measured across an external load of 4.6 kΩ and Ohm's Law (equation (4.1)). This external load was chosen as a consistent point of comparison, given the range of the external loads (1 MΩ–4.6 kΩ) used in this study. Current output was recorded continuously for 4 days with established plants (around two months old) for eight bryoMFCs (four non-sterile and four near-sterile; electronic supplementary material, figure S3) as shown in figures 3*a* and 4*a*. During this period, the average current output was 51.4 ± 7.0 µA m^−2^ and 3.1 ± 0.9 µA m^−2^ for non-sterile and near-sterile systems, respectively.

The non-sterile bryoMFCs were characterized by oscillations with fluctuations between the trough (in the dark) and the peak (in the light) values (figure 3*a*). The average current density in the four non-sterile bryoMFCs was 43.3 ± 7.3 and 64.8 ± 8.0 µA m^−2^ for dark trough and light peak, respectively. For the four near-sterile bryoMFCs, the average current densities for the dark trough and the light peak were 2.3 ± 0.9 and 4.2 ± 1.0 µA m^−2^, respectively (figure 4*a*). Thus, the increase in current density in the light was lower in absolute terms for the near-sterile bryoMFCs, but higher as a fraction of the dark power output (83% compared with 50%) for the non-sterile bryoMFCs. The total charge accumulation after 4 days of continuous running averaged over the four non-sterile bryoMFC reached 18.2 ± 2.3 C m^−2^ (figure 3*c*). By contrast, in the near-sterile bryoMFCs, charge accumulation was only 1.1 ± 0.3 C m^−2^ (figure 4*c*). The current output for the negative controls (i.e. moss-free) for three non-sterile and three near-sterile bryoMFCs over a same period of 4 days was 28.6 ± 4.6 µA m^−2^ and only 2.6 ± 1.3 µA m^−2^, respectively (figures 3*b* and 4*b*). For these negative controls, the total charge accumulation after 4 days of continuous running averaged over the three non-sterile and the three near-sterile bryoMFCs reached 10.3 ± 1.6 C m^−2^ and 0.9 ± 0.5 C m^−2^, respectively (figures 3*d* and 4*d*).

### 2.3. Power density of bryophyte microbial fuel cells

**Figure 5.**
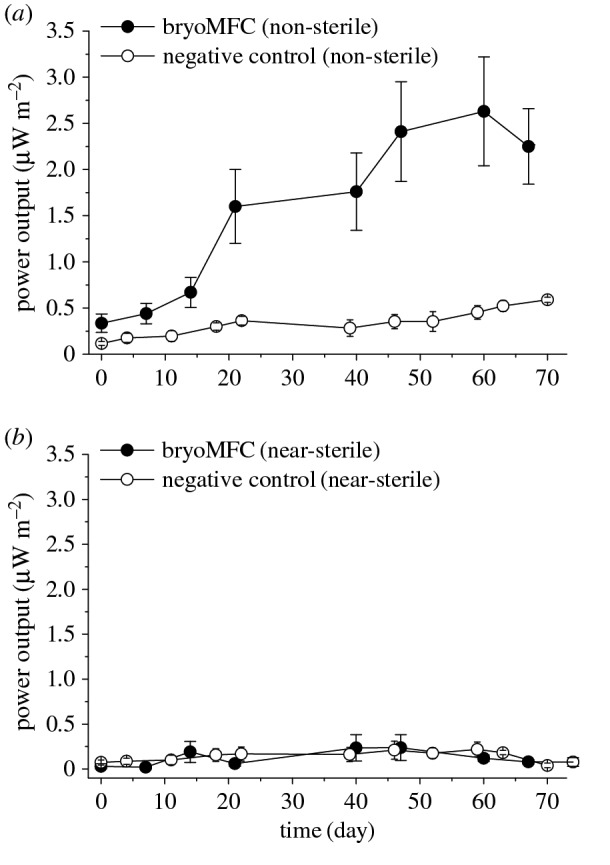
Peak power output for bryoMFCs operating with a novel three-dimensional anodic matrix. The error bars show the standard error (*n* = 4 and *n* = 3 for the negative controls). (*a*) bryoMFC non-sterile device (*b*) bryoMFC near-sterile device.

Power output was determined for the bryoMFCs over periods of 70 days with established *P. patens* plants and negative controls without plants (figure 5). The power density was calculated from the voltage measured across variable external load (1 MΩ–4.6 kΩ) and Ohm's Law (equations (4.1) and (4.2)). The peak power outputs for non-sterile bryoMFCs increased from 0.3 ± 0.1 µW m^−2^ on day 0, to a maximum of 2.6 ± 0.6 µW m^−2^ on day 60 of the experimental run (figure 5*a*, black trace). A representative polarization curve for non-sterile bryoMFCs as recorded at day 67 is shown in the electronic supplementary material, figure S4.

By contrast, the three non-sterile negative controls without plants reached a maximum of 0.6 ± 0.2 µW m^−2^ on day 70, confirming the requirement for plant material in the non-sterile bryoMFC system. The peak power output for near-sterile bryoMFCs showed negligible change over time reaching a maximum around 0.2 ± 0.1 µW m^−2^ on day 47 of the experiment (figure 5*b*). Three negative near-sterile negative controls (plant free) reached an equal power output of 0.2 ± 0.1 µW m^−2^ on day 63.

### 2.4. Current and power density in bryophyte microbial fuel cells operated with environmental samples of moss

**Figure 6.**
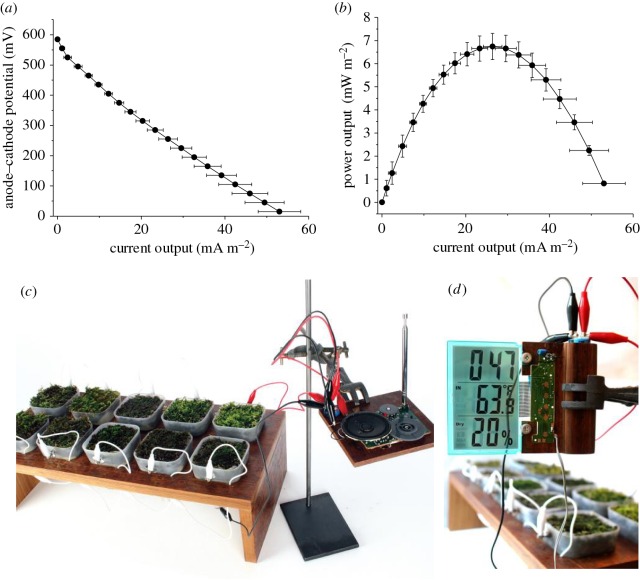
Characterization of the MossFM. The figure shows polarization (*a*) and power (*b*) curves for the bio-electrochemical systems forming the Moss FM (*n* = 10), as well as the actual Moss FM powering a commercial radio (*c*) and a digital LCD environmental sensor (*d*).

The electrochemical characterization (polarization and power curves) of the 10 pots operated with environmental samples containing a mixture of six species of moss is shown in figure 6*a*,*b*. When characterized individually, the average peak power of the 10 pots reached 6.7 ± 0.6 mW m^−2^ with an average maximum current density of 53.0 ± 5.1 mA m^−2^. When the 10 pots were connected together and used to form a demonstration prototype named ‘Moss FM’ (figure 6*c*,*d* and electronic supplementary material, figures S5–S7), a commercial radio (Sony ICF-S22 FM radio, Maplin UK) or an environmental sensor (LCD Desktop Weather Station, Maplin UK) could be powered. To power the radio, the 10 pots were connected in series and used to charge a rechargeable battery with a nominal voltage of 3.6 V (obtained from a Three LED Solar Key Ring Light, Maplin UK). After approximately 10 h of charging, the battery was able to power the radio for about 80 s [29].

To power the environmental sensor, 10 pots were divided into two lots of five. In each lot, the five pots were connect in series and the two lots connected in parallel. This allowed the desktop weather station to be powered directly and continuously for over a week (data not shown).

## 3. Discussion

This study successfully created and characterized a novel three-dimensional anodic matrix, and tested the suitability of mosses to generate electrical output in a MFC using the novel anodic matrix. Five test samples of anodic matrix variant were created with the weight-to-weight ratio of p : C ranging from 40 : 1 to 2 : 1 (electronic supplementary material, figure S2). *Physcomitrella patens* grew on all of these, and consumed carbon dioxide and accumulated biomass at rates similar to moss on a reference sample made of paper only, except for the sample of matrix variant with the highest loading of carbon (p : C ratio 2 : 1), where the plants of *P. patens* looked smaller (figure 2*f*) and accumulated only 2.40 ± 1.23 mg cm^−2^ of dry mass in about 20 days of growth. This value is statistically significantly smaller than the biomass accumulated by the plants grown on the reference sample (7.70 ± 1.00 mg cm^−2^; electronic supplementary material, table S1). The rate of carbon consumption for this sample (17.5 nmol CO_2_ mgChl^−1^ h^−1^ @ 300 µE m^−2^ s^−1^) was also lower than the rate measured for the reference sample (26.0 ± 2.7 nmol CO_2_ mgChl^−1^ h^−1^ @ 300 µE m^−2^ s^−1^; figure 2*g*) but the difference was not statistically significant (electronic supplementary material, table S1). The physiological significance, if any, of this apparent difference is unclear as the sample of matrix variant with the highest loading of carbon occupied a larger volume in the Petri dishes, so the moss might have been physically constrained.

The five samples of matrix variant were as effective as the reference sample (paper only) for retaining water over time (figure 2*i*). By contrast, the five samples were substantially different in electrical resistance. Optimal electrical resistance was seen in the reference sample exclusively made up of carbon fibre (12.0 ± 7.5 Ω) (electronic supplementary material, figure S8). The sample with the lowest loading of carbon fibre (40 : 1) had a resistance 6000 times greater than the reference, while the matrix variant with the highest carbon fibre concentration (2 : 1) had a resistance only four times greater than the carbon fibre reference sample, which was not statistically significantly different (electronic supplementary material, table S1). The three samples with intermediate loading of carbon (p : C ratio 20 : 1, 10 : 1 and 5 : 1) had resistances about 2500, 200 and 100 times larger than the sample of carbon fibre (figure 2*j*). The optimal ratios of p : C (giving the lowest resistance) would be 10 : 1, 5 : 1 and 2 : 1. However, given the requirement for biocompatibility of the various samples, the matrix with the highest loading of carbon (p : C ratio 2 : 1) was unsuitable. For the two remaining samples (p : C ratio 10 : 1, 5 : 1), there was no statistically significant difference in resistance (electronic supplementary material, table S1), so the more moderate loading (10 : 1) was preferred in view of the relative cost of tissue paper and carbon fibre (approx. 1 : 100).

**Figure 7.**
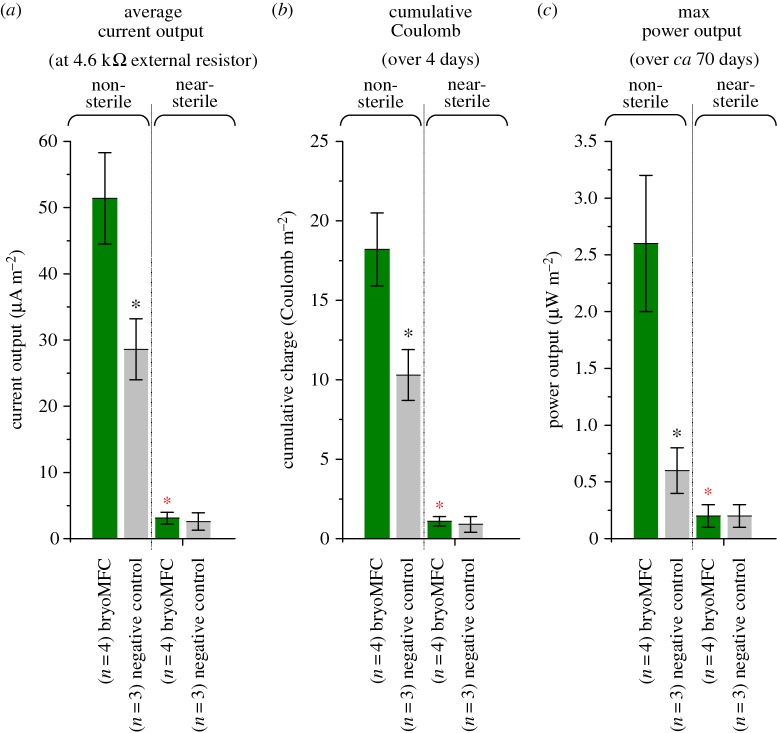
Comparison of the electrical output in bryoMFCs. Comparison of the current output of bryoMFCs (*a*). Comparison of the Coulomb accumulation over 4 days of continuous running of bryoMFCs (*b*). Comparison of the power output of bryoMFCs (*c*). Black asterisk: an ANOVA test found the value of the non-sterile moss-free controls differed significantly from the non-sterile bryoMFCs operated with moss with a *p*-value of less than or equal to 5%. Red asterisk: an ANOVA test found the value of the near-sterile bryoMFCs operated with moss differed significantly from the non-sterile systems operated with moss with a *p*-value of less than or equal to 5%.

The matrix variant with optimal p : C ratio (10 : 1) was used as anodic matrix to test if the bryophyte *P. patens* could be used to generate electrical output in an MFC system. In non-sterile bryoMFCs operated with *P. patens*, the current output (51.4 ± 7.0 µA m^−2^), charge accumulation (18.2 ± 2.3 C m^−2^) and power output (2.6 ± 0.6 µW m^−2^) were statistically different from the non-sterile negative controls lacking plant material (28.6 ± 4.6 µA m^−2^, 10.3 ± 1.6 C m^−2^ and 0.6 ± 0.2 µW m^−2^; electronic supplementary material, table S1; black asterisk, figure 7*a*–*c*). This finding is consistent with published studies where vascular plants and algae have been used to generate electrical power in PMFCs and BPVs, respectively [19,22,23,30,31].

The study successfully compared the electrical output of bryoMFCs run under non-sterile and near-sterile conditions. In the experiments conducted with *P. patens* in non-sterile conditions, oscillation in the current output was clearly seen in every independent replicate (figure 3*a*). The negative controls lacked *P. patens* and no pattern of oscillation in the current output was observed (figure 3*b*).

In the experiments conducted with *P. patens* in near-sterile conditions, small oscillation in the current output was seen in every independent replicate (figure 4*a*). The negative controls lacked *P. patens* and no pattern of oscillation in the current output was observed (figure 4*b*). The oscillations of electrical output in the bryoMFCs further supported the key role of *P. patens* in generating the electrical output. These oscillations suggest a constant phase relationship between the light–dark cycle and current output of the bryoMFCs (figures 3*a* and 4*a*). Current output fluctuation was in phase with the light–dark cycle. A similar behaviour has been observed in BPVs that operated with unicellular photosynthetic microorganisms [21]. The phase relationship in the bryoMFC is presumably because the metabolites which are oxidized to generate the electrical output originate from the moss plants through photosynthesis. Therefore, the production of these compounds would be inherently correlated with photosynthetic metabolism, resulting in an in-phase relationship of light and electrical output. The ratio of the current output for the light peak and the dark trough in near-sterile systems was also observed to be greater than that of non-sterile systems. This reflects the low dark output level in the non-sterile systems, and perhaps also the greater ratio of abundance of plant metabolites available for oxidation to the relatively small microbial populations in near-sterile conditions. Further analyses of plant metabolites and microbial communities are necessary to evaluate critically these observations.

**Figure 8.**
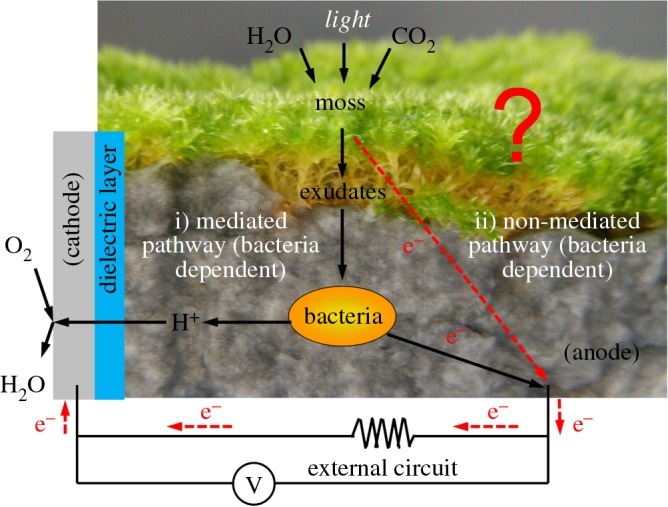
Principle of bryoMFC operation. The electrogenic activity of *P. patens* could go through a (i) mediated (ii) non-mediated pathway. The mediated pathway requires the mediation of anodophilic microbes. The anodic material shown in this figure is the novel three-dimensional matrix.

The matrix was conceived to present advantages over a solid carbon sheet used in previous studies [23,30,32,33]. Given the ability of the novel substrate to be conductive throughout its volume there is greater surface area for interaction between electron donating microorganisms and anode. However, as the filaments of *P. patens* did not penetrate the three-dimensional anodic matrix more than 1–2 mm (figure 8), the full potential of this three-dimensional conductive matrix was probably not completely realized. The use of this novel matrix might yield higher current in devices operated with vascular plants (e.g. PMFC) whose root systems penetrate deeper into their growing substrate and hence increase the rhizosphere surface area.

Analysis of the role of microbes is important as they have been considered essential for achieving high-power output [34]. As mentioned previously, exoelectrogens metabolize organic substrates and transfer electrons to the anode through direct contact or extracellular electron shuttles. In bryoMFCs, these microorganisms may metabolize organic compounds released by bryophytes into the anodic matrix. Naturally forming microbial communities in non-sterile systems (i.e. not inoculated with exoelectrogens) may result in competition for electron donors released by the bryophytes. While competition for plant-derived metabolites may exist, syntrophic interactions between different microbial species are also a possibility [34]. In this study, the peak power output achieved by any of the recorded bryoMFCs operated with *P. patens* was only approximately 3 µW m^−2^ (figure 7*c*), considerably lower than power outputs reported in PMFC (approx. 679 mW m^−2^) [35] and also BPV studies (approx. 100 mW m^−2^) [31]. However, unlike other studies, this investigation was carried out under natural conditions, without the addition of extraneous organic material or inoculation of exoelectrogens to help the formation of anodic microbial communities [36]. This consideration is important as inoculation of exoelectrogenic microbial communities into the anodic region is known to deliver higher power output [36,37]. This study is similar to a previous study by Bombelli *et al*. [38], and focused on the ability of naturally forming microbial communities to generate electrical power. The comparison in figure 7*a*–*c* shows that the current output, charge accumulation and power output for the non-sterile systems (51.4 ± 7.0 µA m^−2^, 18.2 ± 2.3 C m^−2^ and 2.6 ± 0.6 µW m^−2^) were statistically significantly different from the values recorded for the bryoMFCs run under near-sterile conditions (3.1 ± 0.9 µA m^−2^, 1.1 ± 0.3 C m^−2^ and 0.2 ± 0.1 µW m^−2^; electronic supplementary material, table S1; red asterisk, figure 7). By contrast, there were no statistically significant differences among the current output, charge accumulation and power output for the near-sterile systems operated with *P. patens* (3.1 ± 0.9 µA m^−2^, 1.1 ± 0.3 C m^−2^ and 0.2 ± 0.1 µW m^−2^) and their plant free negative control (2.6 ± 1.3 µA m^−2^, 0.9 ± 0.5 C m^−2^ and 0.2 ± 0.1 µW m^−2^; electronic supplementary material, table S1).

This indicates that *P. patens* is unable to generate electrical output independent of microorganisms as depicted in figure 8 (i.e. that the red dotted line is not effective in the systems studied), and bryoMFCs should be operated under non-sterile conditions. It would be interesting to characterize the microbial populations in the anodic region, and whether they change with time, to see if some microbial strains might mediate the electrical property of *P. patens* better than others. The microbial community in the anodic region might have additional useful functions. For example, common soil bacteria such as Sphingomonadales and *Variovorax paradoxus* are known to break down aromatic compounds and inactivate heavy metal (e.g. arsenate, chromate, Hg, Cu, Cd, Zn, Co and Ni), respectively [39]. Thus, the microbial community in the moss rhizosphere could have the potential for bioremediation, giving additional value to the bryoMFC.

The matrix developed in this study was also used to create 10 bryoMFCs operated with an environmental sample containing a mix of six different moss species. The power density with the environmental samples was significantly greater than with *P. patens*. This could be owing to any of a number of factors, including the moss or microorganism species present, or the amount of organic matter associated with the environmental samples on collection. The 10 bryoMFCs were combined into a demonstrative prototype named Moss FM (figure 6*c*,*d* and electronic supplementary material, figure S5–S7) and used to power a commercial radio and an LCD desktop weather station. To the best of our knowledge, the Moss FM described in this study is the first example of bryoMFC able to run a commercial radio (Sony ICF-S22 FM radio, Maplin UK) powered by a battery that was solely charged by moss.

We have shown that: (i) bryophytes (the model moss species *P. patens*, and other environmental samples of moss) can be used in an MFC set-up to generate electrical output in conjunction with microorganisms; (ii) the comparison of near-sterile versus non-sterile bryoMFCs confirmed the importance of microbial populations for transduction of the electrogenic activity; and (iii) the bryoMFCs equipped with environmental sample of moss can be used to power a commercial radio or an environmental sensor.

## 4. Material and methods

### 4.1. Anodic matrix

The composite anodic material used in the bryoMFC system comprised 10 g of tissue paper (Highly Absorbent Fabric, Kimberly-Clark, UK) and 1 g of carbon fibre (CarbonMods, UK). Carbon fibre anodes have been used previously as a three-dimensional flexible network [38] or as a planar sheet of carbon cloth [40]. Here, we separated the strands of the carbon cloth into single fibres and cut the fibres approximately into 4 cm ± 2 cm lengths and created a paper/carbon fibre pulp matrix to allow enhanced contact between moss and the anodic surface. Five test samples of this matrix were generated, with the weight-to-weight ratio p : C ranging from 40 : 1, 20 : 1, 10 : 1, 5 : 1 to 2 : 1. These were tested against a control sample made of paper only (p : C ratio of 1 : 0).

This mixture of paper and carbon fibre was formed using a blender (Philips, HR/2810-A) with the addition of approximately 300 ml of deionized water. The resulting pulp was transferred to a plastic container (85 mm × 65 cm), with a stainless steel bolt (70 mm long, 6 mm ø) acting as electron collector. The matrix was created on the assumption that the thickness (several millimetres) would provide more surface area for improving the contact between moss and anode, and consequently improve electrical output. The matrix variants and the control are shown in the electronic supplementary material, figure S2. To serve as an anodic substrate for a bryoMFC, the matrix variants and the control sample were evaluated as a suitable anodic growth substrate for a bryoMFC, and desirable characteristics such as: (i) biocompatibility, (ii) water retention, and (iii) low electric resistance were investigated.

### 4.2. *Physcomitrella patens* growth on the anodic matrix variants and biomass accumulation

Sections of the samples of composite matrix, approximately 4 cm × 3 cm (12 cm^2^), were placed into 100 mm Petri dishes with 10 ml of BCD medium [27]. The medium does not provide a major source of fixed carbon. Equal amounts of blended filaments of *P. patens* were added to the surface of each sample of matrix variant and grown over 20 days as described in ‘Experimental Set-up’, to observe the biocompatibility of the samples.

Biomass accumulated after 20 days' growth was determined by taking the desiccated moss away from the surface of the matrix variant with metal tweezers and determining its mass using a precision balance (Adam Equipment Co. Ltd., Milton Keynes, UK). Specimens were left in a dry and dark cabinet at room temperature for about a month for desiccation.

### 4.3. Carbon assimilation

Carbon dioxide assimilation was measured using the LI6400-XT open gas exchange system (Li-Cor, Lincoln, NE, USA). Each sample comprised a portion (10 cm^2^) of moss and matrix variant with the weight-to-weight ratio p : C ranging from 40 : 1, 20 : 1, 10 : 1, 5 : 1 to 2 : 1 and 1 : 0. Samples were weighed, placed within the Whole Plant Chamber (LI6400-17; Li-Cor) and illuminated using the RGB Light Source (LI6400-18; Li-Cor). Pure CO_2_ was added through the CO_2_ injection unit of the LI6400-XT to provide inflowing air with a CO_2_ concentration of 400 ppm at a flow rate of 200 µmol s^−1^ and chamber air pressure of 99.7 kPa. Leaf temperature was calculated within the Li-Cor using energy balance techniques and set to be maintained at 20.5°C. Photosynthetic parameters were measured at varying photon flux densities (waveband: 400–700 nm; 10% blue fraction (photon basis)) with total photon flux sequentially decreasing from 300 µE m^−2^ s^−1^ to 0. The chlorophyll content of each moss sample was quantified as described in Porra *et al.* [41]. Finally, the rate of CO_2_ consumption (nmol of CO_2_ h^−1^) of each sample was divided by the corresponding nmol of Chl. In this way, the final figure was expressed as nmol CO_2_ nmolChl^−1^ h^−1^.

### 4.4. Water retention

Water retention in the control sample (p : C ratio 1 : 0) and the five samples of matrix variant (p : C ratios 40 : 1, 20 : 1, 10 : 1, 5 : 1 and 2 : 1) was compared by adding 50 ml deionized water to 10 g of dry mass of the six samples. The samples (60 g total mass) were kept at 30°C, and the mass determined every 3–5 h. Results are displayed in figure 2*i* as percentage of water mass remaining.

### 4.5. Electrical resistance

This was measured across the control sample (p : C ratio 1 : 0), the five samples of matrix variant (p : C ratios 40 : 1, 20 : 1, 10 : 1, 5 : 1 and 2 : 1) and a planar sheet of carbon fibre (p : C ratio 0 : 1).The experiment was conducted by placing 10 g (dry mass) of sample into a plastic vessel (110 mm (W) × 75 mm (L) × 50 mm (H)). Two strips of stainless steel (75 mm (L) × 10 mm (W)) were placed onto the sample. The space separating the strips of stainless steel was kept at 90 mm. A force of approximately 5 N cm^−2^ was imposed onto each strip of stainless steel. Electrical wires attached to the strips were connected to a digital multimeter (UNI-T, UT70B). Resistance was recorded after adding 50 g of deionized water for each sample.

### 4.6. Bryophyte microbial fuel cell device construction

The eight identical bryoMFCdevices used were constructed using a plastic chamber (110 mm (W) × 75 mm (L) × 50 mm (H)) as shown in figure 1*c*,*d*. For each device, an anode was made from the matrix variant as described below.

The anode was then fastened into the box and placed in physical contact with a stainless steel screw (70 mm long, 6 mm ø), functioning as the anodic electron collector. When in place, the anode was watered with 50 ml of deionized water. For the anode, the chosen ratio p : C was 10 : 1. The cathode consisted of carbon paper, loaded with Pt (3 mg loading per cm^2^, Alfa Aesar) as catalyst. To expose the catalyst to oxygen, the catalyst was held adjacent to a small hole (7 mm Ø) on the side of the container with duct-tape (Scapa, UK), while the anodic matrix variant (anode) was separated from the catalyst by several sheets of tissue paper (Highly Absorbent Fabric, Kimberly-Clark, UK). The container was dried and then autoclaved. For devices under near-sterile conditions, watering was carried out using a silicone tube with a filter added following autoclave sterilization.

Inoculation of the device was carried out in a similar manner as described in ‘Experimental set-up’ and 90 ml of BCD medium was added with moss filaments. For the control experiments, the same amount of BCD medium without moss filaments was added.

### 4.7. Experimental set-up

A culture of filamentous *P. patens* was propagated on plates covered with cellophane as described by Harrison *et al.* [27]. All cultures were maintained at 20 ± 2°C under moderate light (approx. 25 W m^−2^) in a 24 h light cycle (12 h light/dark) under near-sterile conditions. Cultures were passaged weekly and the concentration was monitored by spectrophotometric determination of chlorophyll extracted in 99.8% (v/v) methanol (Sigma-Aldrich, Gillingham, UK) as described by Porra *et al.* [41] prior to inoculation on the anodic surface (20 nmol Chl per chamber). The inoculum of moss culture (approx. 5 ml) was allowed to settle and attach to the anodic substrate material under static conditions for 12 h. Unless stated otherwise, experiments were conducted with the lid closed. Individual chambers were connected to an external load (1 MΩ) for 7 days to allow stabilization prior to measurement. To normalize the performances of the devices to differences in culture growth rate, mosses were grown for 8 days before the start of recording. Polarization curves were performed every 7 days of operation. The light falling on the bryoMFCs during the light phase (16 h per day) averaged 120 µE m^−2^ s^−1^, with peaks over 300 µE m^−2^ s^−1^. The light phase was a combination of natural and artificial light shown by the yellow background in figures 3 and 4. The negative controls (moss-free) were run under the same conditions used for the bryoMFCs operated with moss except that the negative controls were wrapped with aluminium foil to prevent the proliferation of autotrophic organisms (e.g. algae).

### 4.8. Analytical techniques

Polarization curves were generated for every bryoMFC operated with *P. patens* and the relevant negative control by recording the cell voltage (*V*) under pseudo-steady state conditions [7] with variable external loads (*R*_ext_) from 1 MΩ to 4.7 kΩ (1 MΩ; 470, 200, 100, 47, 15, 10 and 4.6 kΩ), and plotting the cell voltage as a function of current density (current per unit anodic plant growth area (APGA)). Current was calculated from Ohm's Law as in the following equation:

4.1 $$I=\frac{V}{R_{\mathrm{ext}}}.$$

Based on the polarization curves, power curves were drawn for each system by plotting power per unit APGA or power density as a function of current density. Power *P* was calculated as shown in the following equation:

4.2 $$P=\frac{V^{2}}{R_{\mathrm{ext}}}.$$

The power density curves were further used to determine the average maximum power output for the bryoMFC and the negative control.

Polarization curves were generated for the bryoMFC containing environmental moss isolates using an Autolab PGSTAT12 (Metrohm/EcoChimie, The Netherlands) connected to a computer. Following stabilization at the open circuit potential, polarization and power curves were obtained by scanning the applied voltage from its open circuit potential to 0 volts at a scan rate of 1 mVs^−1^ [42].

### 4.9. Statistical analysis

One-way analysis of variance (ANOVA) was used to determine whether there were any significant differences between the means of independent (unrelated) groups of data.

When the *p*-value is greater than 0.05, there is no statistically significant difference between group means. The complete results obtained from the ANOVA tests run in this study are shown in the electronic supplementary material, table(s) S1. The results were calculated using online software [43].

### 4.10. Construction of the moss pots used to form the Moss FM

The matrix developed in this study was also used as an anodic matrix for testing the electrogenic activity of environmental samples of moss. A mix of six species (*Amblystegium serpens*, *Brachythecium rutabulum*, *Bryum capillare*, *Bryum dichotomum*, *Ceratodon purpureus* and *Syntrichia ruralis*) was obtained from the roof of the bicycle shed in the Department of Biochemistry, Downing Site, Cambridge, UK (electronic supplementary material, figure S5). Ten identical bio-electrochemical systems (bryoMFCs) to create the Moss-FM were constructed using a plastic chamber (70 mm (W) × 70 mm (L) × 60 mm (H)) (electronic supplementary material, figures S6 and S7). The apparatus was designed and constructed by Fabienne Felder. For each device, an anode was made from the matrix (electronic supplementary material, figure S2) as described above. The anode was then fastened into the box and placed in physical contact with a stainless steel mesh and secured to the side of the pot by a stainless steel screw (40 mm long, 5 mm ø), functioning as the anodic electron collector. When in place, the anode was watered with a further 100 ml of tap water.

The cathode consisted of carbon paper, loaded with Pt (3 mg loading per cm^2^, Alfa Aesar) as a catalyst. To expose the catalyst to oxygen, the catalyst was held adjacent to a small hole (20 mm Ø) on the bottom of the container with a plastic frame, soft gaskets and four stainless steel screws. The anodic matrix (anode) was separated from the catalyst by several sheets of tissue paper (Highly Absorbent Fabric, Kimberly-Clark, UK). Inoculation of the device was conducted by adding approximately 50 cm^2^ of an environmental sample of moss picked from the bicycle shed in the Department of Biochemistry, University of Cambridge, UK. The environmental samples of moss were kept in the 10 systems for few weeks before characterization of current and power output. The 10 devices constituting the Moss FM radio were kept in the same experimental conditions described for the bryoMFCs and watered with approximately 100 ml of tap water added every other day. To power the radio, 10 pots were connected in series and used to charge a rechargeable battery with a nominal voltage of 3.6 V. The battery was previously discharged by connecting it across an external resistor of 100 Ω for about 24 h. After that, it was confirmed that the discharged battery was unable to power the radio by connecting the battery to it. After approximately 10 h of charging, the battery was able to power the radio for about 80 s [29].

## Supplementary Material

1)The ESM. This file includes: -SF1. Established shoots (several months old) of P. patens taken from a bryoMFC system. -SF2. Six test samples of the anodic matrix variant. -SF3. BryoMFC systems with P. patens. (a) four non sterile and (b) four sterile. -SF4. Bicycle shed in the Department of Biochemistry in Cambridge where wild moss was taken to construct the Moss FM. -SF5. Details of the bioelectrochemical systems (bryoMFC) to form Moss FM. -SF6. Cartoon describing Moss FM. -SF7.The planar sheet of carbon fibre.

## Supplementary Material

Supplementary Table 1. One-way analysis of variance is calculated for Rate of carbon consumption, Accumulation of biomass, Electric resistance, Biotic bryoMFC vs abiotic bryoMFC, Biotic non sterile bryoMFC vs biotic sterile bryoMFC and Biotic sterile bryoMFC vs abiotic sterile bryoMFC.

## Supplementary Material

Supplementary Table 2. BryoMFC manuscript - Bio mass accumulation on Composite Material - Fig2h

## Supplementary Material

Supplementary Table 3. BryoMFC manuscript - Carbon-paper conductivity - Fig2j

## Supplementary Material

Supplementary Table 4. BryoMFC manuscript - Carbo-paper water retention - Fig2i

## Supplementary Material

Supplementary Table 5. BryoMFC manuscript - Current Coulomb and Power - Fig7

## Supplementary Material

Supplementary Table 6. BryoMFC manuscript - Dark-Light 4 days NonSterile - Fig3a and c

## Supplementary Material

Supplementary Table 7. BryoMFC manuscript - Dark-Light 4 days NonSterile Negative Control - Fig3b and d

## Supplementary Material

Supplementary Table 8. BryoMFC manuscript - Dark-Light 4 days Sterile - Fig4a and c

## Supplementary Material

Supplementary Table 9. BryoMFC manuscript - Dark-Light 4 days Sterile Negative Control - Fig4b and d

## Supplementary Material

Supplementary Table 10. BryoMFC manuscript - MossFM - Fig6

## Supplementary Material

Supplementary Table 11. BryoMFC manuscript - Power over time - Fig5

## Supplementary Material

Supplementary Table 12. BryoMFC manuscript - Rate of CO2 consumption - Fig2g

## Supplementary Material

Bombelli data.zip

## Data accessibility

Our data are deposited and available at the RSOS website.
