# Supplementary material for: Electrical output of bryophyte microbial fuel cell systems is sufficient to power a radio or an environmental sensor
Source: R Soc Open Sci. 2016 Oct 26;3(10):160249. doi: 10.1098/rsos.160249 (PMC5098967; doi:10.1098/rsos.160249)
Supplement: 1)The ESM. This file includes: -SF1. Established shoots (several months old) of P. patens taken from a bryoMFC system. -SF2. Six test samples of the anodic matrix variant. -SF3. BryoMFC systems with P. patens. (a) four non sterile and (b) four sterile. -SF4. Bicycle shed in the Department of Biochem [file rsos160249supp1.pdf]

Title:

Electrical output of bryophyte microbial fuel cell (bryoMFC) systems is sufficient to power a radio or an environmental sensor

(SUP. FIGUREs)

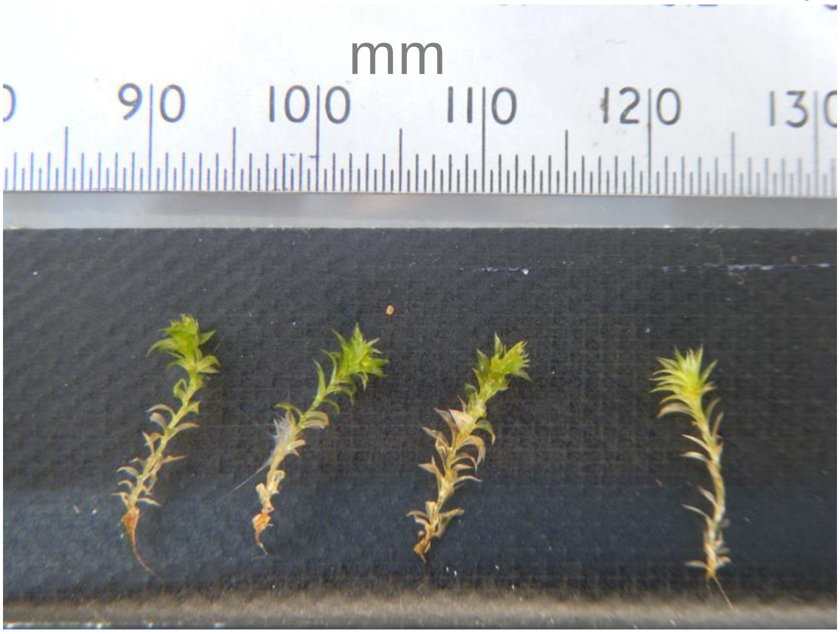

SF1.

Established shoots (several months old) of *P. patens* taken from a bryoMFC system.

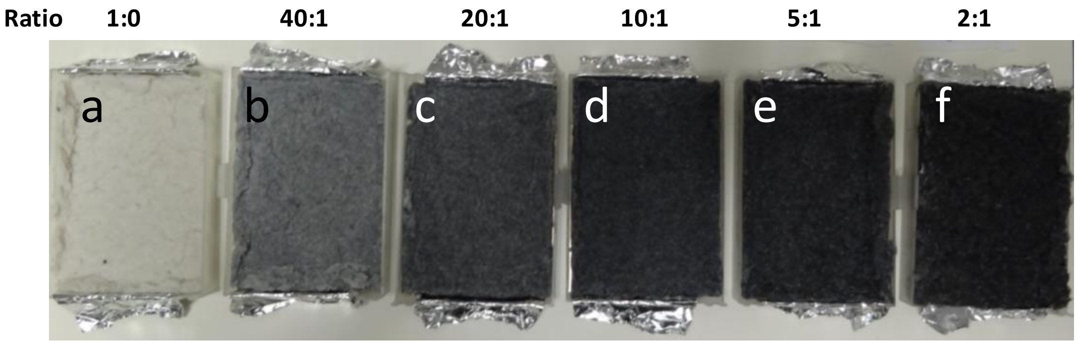

SF2.

Six test samples of the anodic matrix variant. The test **(a)** is a control sample made by paper only (p:C ratio of 1:0). The other five tests the ratio of the paper to carbon fibre (p:C) as weight-to-weight was varied from 40:1 (**b**), 20:1 (**c**), 10:1 (**d**), 5:1 (**e**) to 2:1 (**f**).

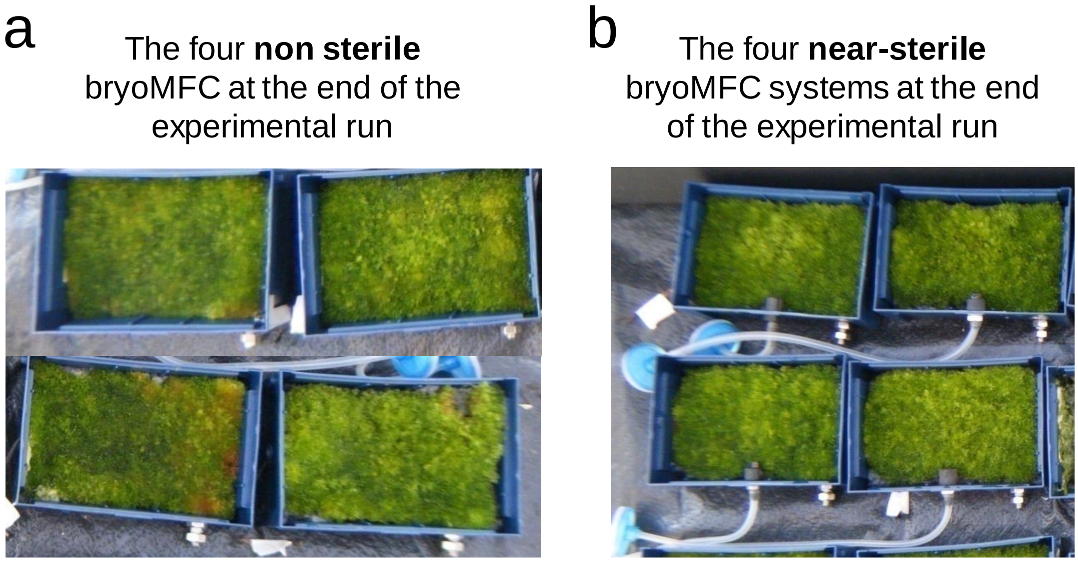

SF3. The eight bryoMFC systems with *P. patens*. Figure shows (**a**) four non sterile and (**b**) four near-sterile systems.

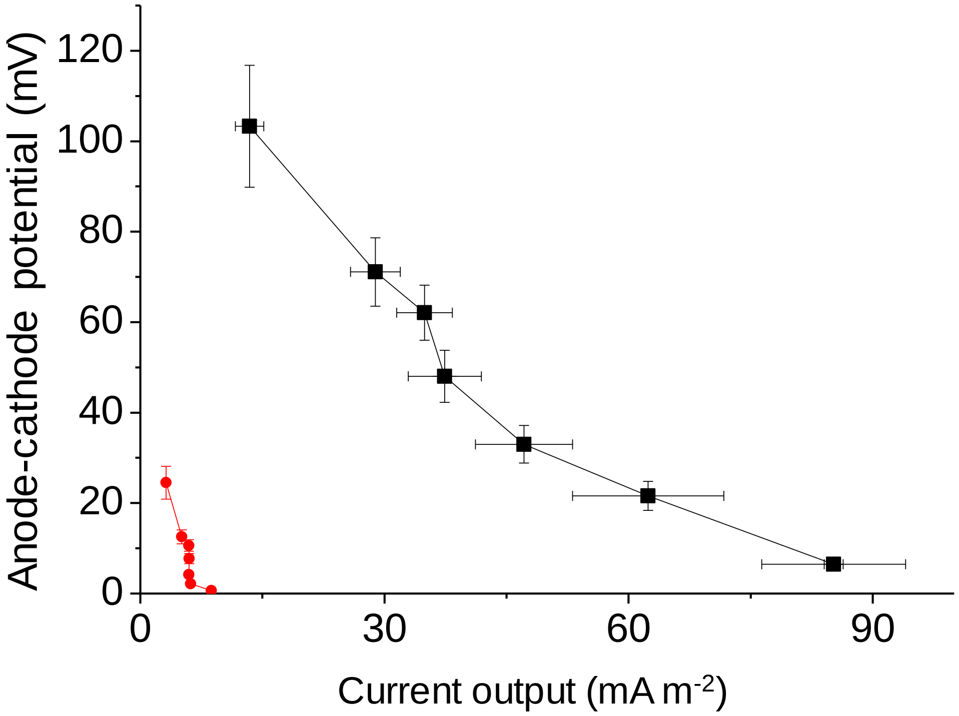

SF4. Representative polarization curve for bryoMFCs operated with *P. patens* as recorded at day 67. The black and red plots show data for non-sterile and near-sterile systems respectively. The data correspond to internal resistances for the non-sterile and near-sterile samples of 1.32 and 4.39 kΩ respectively.

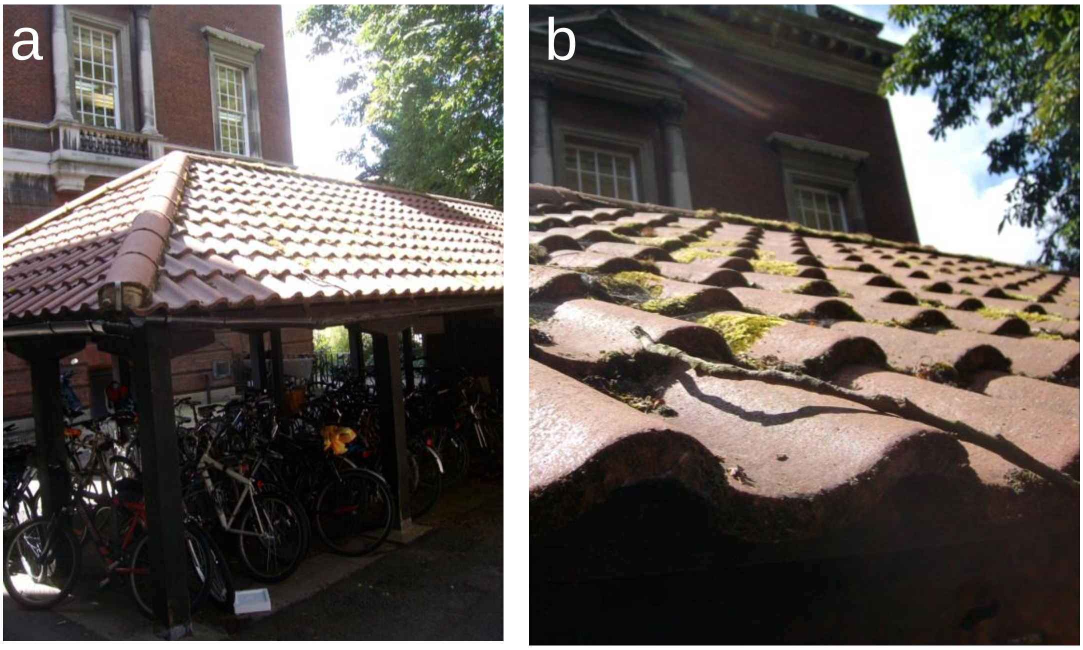

SF5. Bicycle shed in the Department of Biochemistry in Cambridge where environmental samples of moss were taken to construct the Moss FM. (**a**) overview of the bike shed, (**b**) details of the moss attached to the roof-tiles.

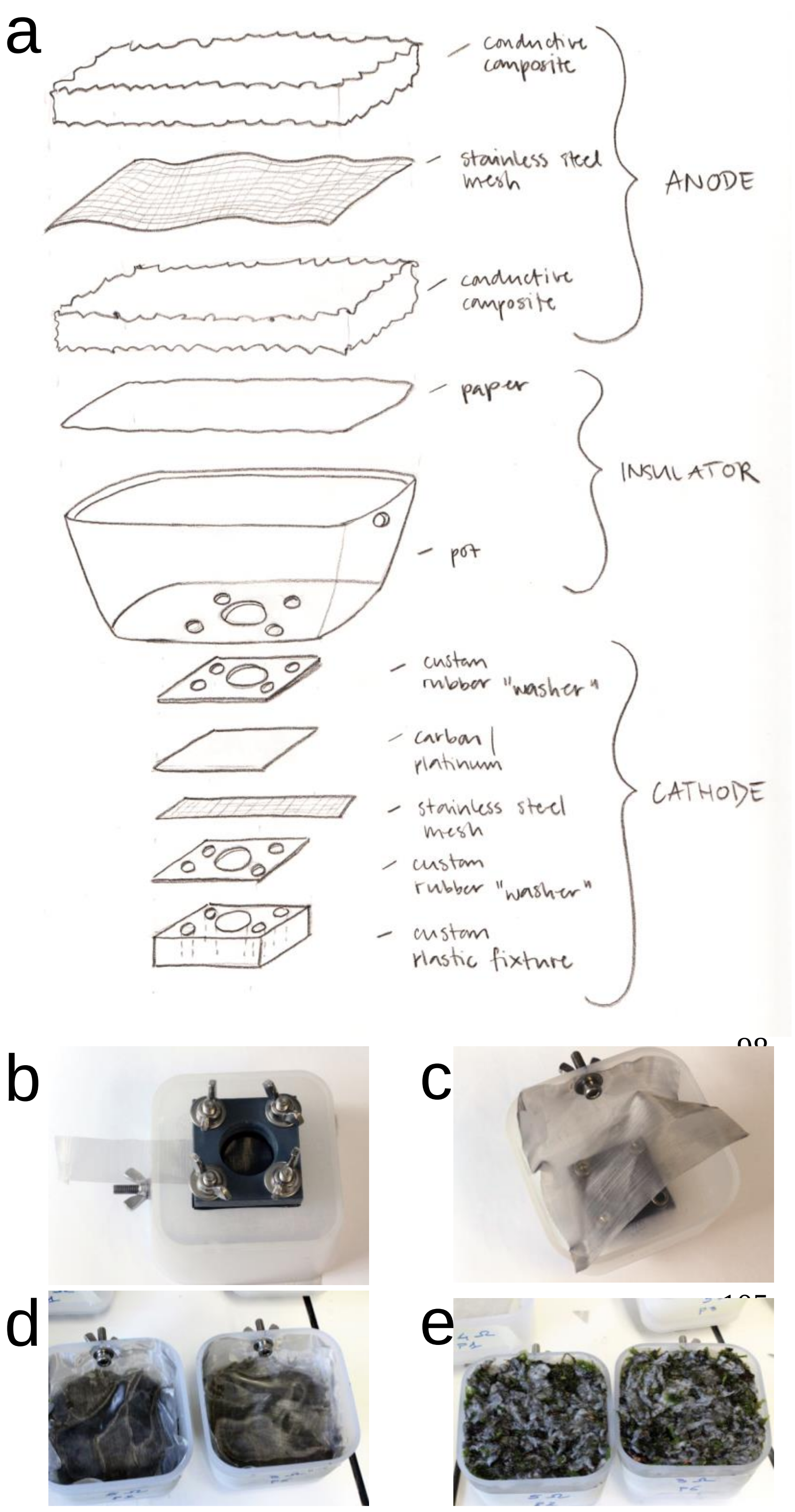

SF6. Details of the bioelectrochemical systems (bryoMFC) created by Fabienne Felder to form Moss FM. (**a**) Schematic of the internal structure where: anode was made by a layer of stainless steel mesh sandwiched between the conductive matrix variant (carbon fibre and cellulose). Attached to the pot with a stainless steel screw. The insulator was made by a layer of paper separates the anodic part from the cathode. The cathode was made by a plate of carbon and platinum overlain with stainless steel mesh and sandwiched between two layers of rubber and held in place with a plastic frame and stainless steel screws. **(b-e)** actual pictures and details of the moss pots.

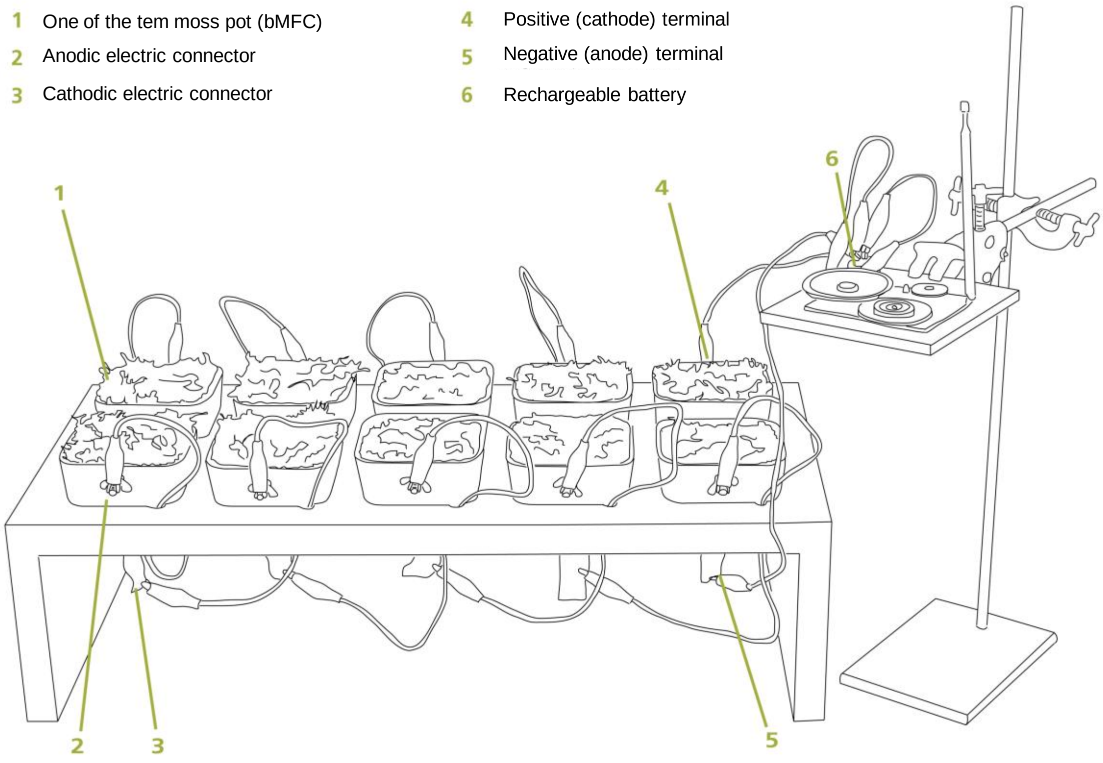

SF7. Cartoon describing Moss FM

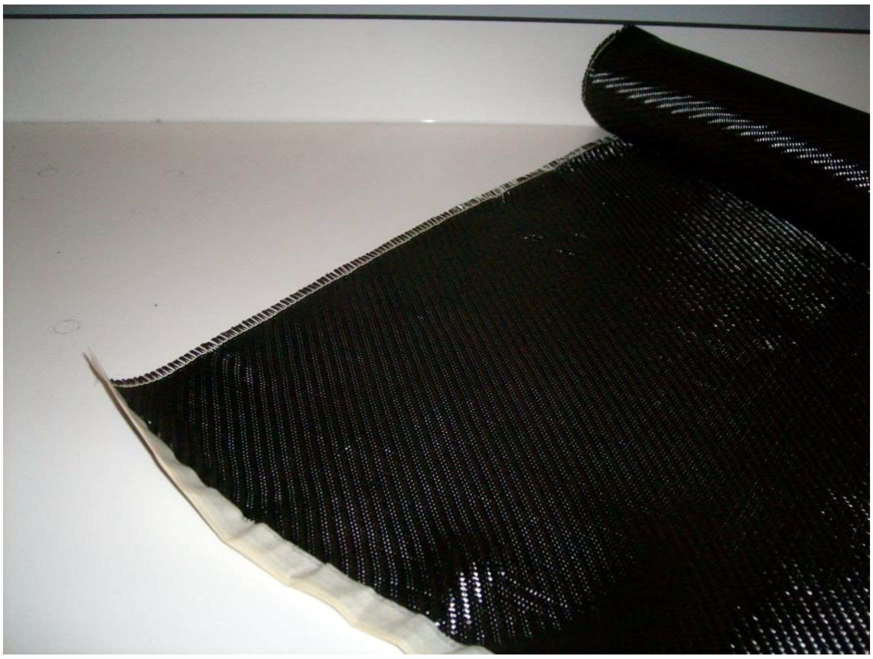

SF8.
The planar sheet of carbon fibre.

Supplementary Table(s) 1
Given the mean, standard deviation, and (n) the one-way analysis of variance is calculated by an ANOVA test where:
SS: sums of squares;
df: degrees of freedom;
MS: mean squares;
F and p-values.

Rate of carbon consumption
Comparison between the rate of carbon consumption for plants grown on matrix variant with the highest loading of carbon (p:C ratio 2:1) versus the rate of carbon consumption of plants grown on reference sample (p:C ratio 1:0).
For plants grown on matrix variant (p:C ratio 2:1): 17.5 nmol CO_2 $mgChl^{-1}$ h^{-1} (n=1)
For plants grown on matrix variant (p:C ratio 1:0): 26.0±2.7 nmol CO_2 $mgChl^{-1}$ h^{-1} (n=3)

	SS	df	MS	F	**p**
Between:	54.188	1	54.188	7.433	**0.112**
Within:	14.58	2	7.29		

Accumulation of biomass

Comparison between accumulation of biomass for plants grown on matrix variant with the highest loading of carbon (p:C ratio 2:1) versus the biomass accumulated for plants grown on reference sample (p:C ratio 1:0).
For plants grown on matrix variant (p:C ratio 2:1): 2.40±1.23 mg cm^{-2} (n=3)
For plants grown on matrix variant (p:C ratio 1:0): 7.70±1.00 mg cm^{-2} (n=3)

	SS	df	MS	F	**p**
Between:	42.135	1	42.135	33.535	**0.004**
Within:	5.026	4	1.256		

Electric resistance

Comparison between the electric resistance for the sample of matrix variant with the highest loading of carbon (p:C ratio 2:1) versus the positive control (i.e., sample exclusively made up of carbon fibre) (p:C ratio 0:1).
For the sample of matrix variant (p:C ratio 2:1): 39.0±16.1 Ω
For the positive control (p:C ratio 0:1): 12.0±7.5 Ω

	SS	df	MS	F	**p**
Between:	1093.5	1	1093.5	6.933	**0.054**
Within:	630.92	4	157.73		

Comparison between the electric resistance for the sample of matrix variant with the loading of carbon (p:C ratio 10:1) versus the sample of matrix variant with the loading of carbon (p:C ratio 5:1).
For the sample of matrix variant (p:C ratio10:1): 200.0±80.0 Ω
For the positive control (p:C ratio 5:1): 105.0±70.0 Ω

	SS	df	MS	F	**p**
Between:	13537.5	1	13537.5	2.396	**0.197**
Within:	22600	4	5650		

Biotic bryoMFC *vs* abiotic bryoMFC (non sterile systems)

Comparison between the average current output, charge accumulation and max power output of biotic and abiotic non sterile systems.
The average of current output for the biotic non sterile systems was 51.4±6.9 µA m^{-2}
The average of current output for the abiotic non sterile systems was 28.6±4.6 µA m^{-2}

	SS	df	MS	F	**p**
Between:	891.154	1	891.154	24.066	**0.004**
Within:	185.150	5	37.030		

The charge accumulation for the biotic non sterile systems was 18.2±2.3 C m^{-2}
The charge accumulation for the biotic non sterile systems was 10.3±1.6 C m^{-2}

	SS	df	MS	F	**p**
Between:	106.989	1	106.989	25.486	**0.004**
Within:	20.99	5	4.198		

The average of the max power output for the biotic non sterile systems was 2.6±0.6 µW m^{-2}
The average of the max power output for the biotic sterile systems was 0.2±0.1 µW m^{-2}

	SS	df	MS	F	**p**
Between:	6.857	1	6.857	29.557	**0.003**
Within:	1.16	5	0.232		

Biotic non sterile bryoMFC *vs* biotic sterile bryoMFC

Comparison between the average current output, charge accumulation and max power output of biotic non sterile and biotic sterile systems.
The average of current output for the biotic non sterile systems was 51.4±6.9 µA m^{-2}
The average of current output for the biotic sterile systems was 3.1±0.9 µA m^{-2}

	SS	df	MS	F	**p**
Between:	3999.24	1	3999.24	138.43	**0.000**
Within:	144.45	5	28.89		

The charge accumulation for the biotic non sterile systems was 18.2±2.3 C m^{-2}
The charge accumulation for the biotic sterile systems was 1.1±0.3 C m^{-2}

	SS	df	MS	F	**p**
Between:	438.615	1	438.615	163.054	**0.000**
Within:	10.76	4	2.69		

The average of the max power output for the biotic non sterile systems was 2.6±0.6 µW m^{-2}
The average of the max power output for the biotic sterile systems was 0.2±0.1 µW m^{-2}

	SS	df	MS	F	**p**
Between:	8.64	1	8.64	46.703	**0.002**
Within:	0.74	4	0.185		

Biotic sterile bryoMFC *vs* abiotic sterile bryoMFC

Comparison between the average current output, charge accumulation and max power output of biotic and abiotic sterile systems.

The average of current output for the biotic sterile systems was 3.1±0.9 µA m^{-2}

The average of current output for the abiotic sterile systems was 2.6±1.3 µA m^{-2}

	SS	df	MS	F	**p**
Between:	0.375	1	0.375	0.300	**0.613**
Within:	5.0	4	1.25		

The charge accumulation for the biotic sterile systems was 1.1±0.3 C m^{-2}

The charge accumulation for the abiotic sterile systems was 0.9±0.5 C m^{-2}

	SS	df	MS	F	**p**
Between:	0.06	1	0.60	0.353	**0.584**
Within:	0.680	4	0.170		

The average of the max power output for the biotic sterile systems was 0.2±0.1 µW m^{-2}

The average of the max power output for the abiotic sterile systems was 0.2±0.1 µW m^{-2}

	SS	df	MS	F	**p**
Between:	0.00	1	0.00	0.00	**na**
Within:	0.04	4	0.01		
